# The dual effects of root-cap exudates on nematodes: from quiescence in plant-parasitic nematodes to frenzy in entomopathogenic nematodes

**DOI:** 10.1093/jxb/eru345

**Published:** 2014-08-27

**Authors:** Ivan Hiltpold, Geoffrey Jaffuel, Ted C. J. Turlings

**Affiliations:** ^1^Division of Plant Sciences, University of Missouri, 205 Curtis Hall, Columbia, MO 65211-7020, USA; ^2^FARCE laboratory, University of Neuchâtel, Emile-Argand 11, 2000 Neuchâtel, Switzerland

**Keywords:** Below-ground food web, biological control, entomopathogenic nematode (EPN), *Heterodera glycine*, *Heterorhabditis bacteriophora*, *Meloidogyne incognita*, plant defence, plant-parasitic nematode, root-cap exudate.

## Abstract

To defend themselves against herbivores and pathogens, plants produce numerous secondary metabolites, either constitutively or *de novo* in response to attacks. An intriguing constitutive example is the exudate produced by certain root-cap cells that can induce a state of reversible quiescence in plant-parasitic nematodes, thereby providing protection against these antagonists. The effect of such root exudates on beneficial entomopathogenic nematodes (EPNs) remains unclear, but could potentially impair their use in pest management programmes. We therefore tested how the exudates secreted by green pea (*Pisum sativum*) root caps affect four commercial EPN species. The exudates induced reversible quiescence in all EPN species tested. Quiescence levels varied with the green pea cultivars tested. Notably, after storage in root exudate, EPN performance traits were maintained over time, whereas performances of EPNs stored in water rapidly declined. In sharp contrast to high concentrations, lower concentrations of the exudate resulted in a significant increase in EPN activity and infectiousness, but still reduced the activity of two plant-parasitic nematode species. Our study suggests a finely tuned dual bioactivity of the exudate from green pea root caps. Appropriately formulated, it can favour long-term storage of EPNs and boost their infectiousness, while it may also be used to protect plants from plant-parasitic nematodes.

## Highlight text

Plant defences against root antagonists invigorate root protagonists. The same root-cap exudate impairs the mobility of plant-parasitic nematodes and increases the performances of insect-killing nematodes.

## Introduction

The estimated production of over 200,000 different compounds (Barber and Martin, 1976) ranks plant’s secondary metabolites among the most diverse functional chemicals in the biosphere. Thousands of papers have been devoted to the description of ecological and evolutionary processes mediated by plant secondary metabolites (Dudareva *et al.*, 2013). Stahl (1888) was one of the first to suggest that these metabolites are involved in plant defences and they have since been proposed to contribute as an important selecting force in the interaction between plants and herbivores (Ehrlich and Raven, 1964). Historically, work on chemical plant defences has focused on foliar parts of plants (Hartmann, 2007) and roots have been mostly ignored (Hunter, 2001). Yet up to 20% of the photosynthetic fixed carbon is exuded via belowground tissues as secondary metabolites (Barber and Martin, 1976) and these exudates shape rhizospheric interactions (e.g. Hiltpold and Turlings, 2012; Rasmann *et al.*, 2012*a* and references therein; Erb *et al.*, 2013).

In the context of plant and plant-parasitic nematode interactions, root exudates are often beneficial to the plant antagonists, as they may use plant secondary metabolites to locate host plants (Prot, 1980; Rolfe *et al.*, 2000; Curtis *et al.*, 2009; Reynolds *et al.*, 2011). In addition to attracting nematodes, root exudates also trigger egg hatching in certain plant-parasitic nematode species (e.g. Perry and Clarke, 1981; Dennijs and Lock, 1992; Perry and Gaur, 1996; Gaur *et al.*, 2000; Wesemael *et al.*, 2006; Pudasaini *et al.*, 2008; Khokon *et al.*, 2009; Oka and Mizukubo, 2009). Yet root exudates can also protect roots against plant-parasitic nematodes. For instance, metabolites exuded from the root-cap cells of legumes and maize (*Zea mays*) slow down movement in plant-parasitic nematodes, sometimes resulting in a state of quiescence, reducing the ability of the nematodes to infect the plant (Zhao *et al.*, 2000). This apparent defence mechanism has been found in several plant species and is usually reversible (Hubbard *et al.*, 2005).

In addition to plant-parasitic nematodes, root exudates have also been reported to affect entomopathogenic nematodes (EPNs), plant protagonists in the rhizosphere. Rasmann *et al.* (2005) found that insect herbivory on maize roots induces the emission of volatile secondary metabolites that can attract the EPN *Heterorhabditis megidis* Poinar, Jackson and Klein. A similar phenomenon has since been shown for belowground interactions between plants and EPNs in other systems involving cultivated plants (Ali *et al.*, 2010; Hiltpold *et al.*, 2010c; Turlings *et al.*, 2012), as well as in wild ecosystems (Rasmann *et al.*, 2011). As EPNs are obligate parasites that kill their insect host in a very short period of time (Dillman *et al.*, 2012), they are considered as very potent biological control agents in several cropping systems (Grewal *et al.*, 2005). Hubbard *et al.* (2005) found that green pea (*Pisum sativum*) root cap exudate induces quiescence in the EPN *Steinernema feltia* Filipjev, implying that this putative plant defence strategy may impair EPN efficacy near root tips, where many herbivores prefer to feed.

With the current study we aimed at assessing the effect of such exudate on four commercial EPN species. As different crop varieties can greatly vary in defensive metabolite patterns (Takabayashi *et al.*, 1991; Loughrin *et al.*, 1995; Wooley *et al.*, 2007; Erb *et al.*, 2011), we tested the exudate of a number of different pea cultivars for their potency in inducing quiescence in EPNs. In addition, to evaluate the impact of the root-cap exudate on the quality of stored EPNs over time, we performed a series of experiments to assess EPN infectiousness, mobility, and lipid content after recovery from induced quiescence. We also examined to what extent diluting the concentration of the exudate affects quiescence induction and whether heat or cold treatment has an effect on exudate activity.

## Materials and methods

### Plant material

*Pisum sativum* L. cv. Lancet seeds (Wyss Samen und Planzen AG, Switzerland) were sterilized in 95% ethanol for 5min. Seeds were then rinsed and immersed in pasteurized distilled water for 12h. Imbibed seeds were germinated for 3 days at 25°C in the dark in plastic boxes (15×13.5×5cm) on a 1.5cm layer of 1.0% PhytoAgar (Duchefa Biochemie, The Netherlands).

### Root-cap exudate collection

Exudates were collected from 15 pea germinates by arranging them on a Teflon plate at the periphery of a 1ml drop of ultra-pure water, with only the tips (~5mm) submerged. After 2min of immersion, the liquid was gently agitated in order to disperse the border cells and the associated exudate. The solution obtained was then centrifuged at 4000 *g* for 10min in a 1.5ml centrifuge tube (Vaudaux-Eppendorf AG, Switzerland), resulting in a pellet of the suspended border cells. The supernatant, defined as root-cap exudate, was collected. Following this technique, exudates from several hundred of pea germinates were collected, and pooled in one solution. To facilitate the experimentation, this last solution was split into 7ml amber vials (Suppelco, Sigma-Aldrich Chemie GmbH, Switzerland) before being stored at –20°C.

### Exudate-induced quiescence and recovery of EPNs

The pea root-cap exudate was tested on the following EPN species: *Heterorhabditis bacteriophora* Poinar (Rhabditida: Heterorhabditidae), *Heterorhabditis megidis* Poinar, Jackson and Klein (Rhabditida: Heterorhabditidae), *Steinernema feltiae* Filipjev (Rhabditida: Steinernematidae), and *S. carpocapsae* Weiser (Rhabditida: Steinernematidae), following the methodology by Hubbard *et al.* (2005). *H. bacteriophora*, *S. feltiae*, and *S. carpocapsae* were provided by Landi-Reba AG (Switzerland), and *H. megidis* by Becker Underwood (UK).

Briefly, ~1000 active infective juveniles of each EPN species were taken from batches freshly hatched from *Galleria mellonella* L. (Lepidoptera: Galleridae). EPNs were suspended in water and aliquots of 30 infective juveniles in 50 µl of water were pipetted into 36 wells of a 96-well plate (BD Biosciences, CA, USA) and 175 µl of root cap exudate was added to each EPN suspension. After 12h, using a dissecting microscope, the number of quiescent EPN-infective juveniles was evaluated by direct counting of individuals exhibiting, or not exhibiting, an active sinusoidal form and movement. Instead of root-cap exudate, 175 µl of water was pipetted into the wells of the control plates. The experiment was repeated 10 times for each EPN species.

To evaluate EPN recovery from exudate-induced quiescence, 125 µl was pipetted out of the wells, leaving settled EPN in the bottom, and replaced with the same volume of water. After 12h, resumption of EPN activity was evaluated using a dissecting microscope. To test if recovered EPNs were still infectious, five times 10 individuals per species per replicate were sampled and applied on top of *G. mellonella* larvae that had been individually placed in wells of a 24-well plate. The baits were then covered with 10% moist sand (white sand, Migros, Switzerland) and stored at 25°C in the dark. EPN infection was assessed after 48h.

### Quiescence induction by the root exudates of various green-pea cultivars

Following the procedures described above, eight additional cultivars (cv. Kelvin, cv. Kelvin Sprinter, cv. Merveilles précoces, and cv. Sprint, Wyss Samen und Planzen AG, Switzerland; cv. Exquis, cv. Surab, cv. Picolo, and cv. Carmini, Landi-Reba AG, Switzerland) were tested for their ability to induce quiescence in *H. bacteriophora*. Water was used as a control and the EPNs were kept in the respective solutions for 12h before the number of quiescent nematodes was recorded. Each test was repeated 10 times.

### Effect of root-cap exudate on the quality of EPNs over time

Either 41.25ml of root-cap exudate from green pea cv. Lancet or the same amount of water (control) were poured into 50ml culture flasks (BD Biosciences, CA, USA), and 7.5ml of water containing 20 000 *H. bacteriophora* infective juveniles were added. The flasks were then stored at room temperature (25°C) in the dark. Every third day, starting 12h after the induction of quiescence, the infectiousness, mobility, and lipid content of the nematodes from both solutions were assessed for 18 days.

#### EPN infectiousness 

EPN infectiousness was measured using the five-on-one sand well bioassay (modified after Grewal, 2005): In a 24-well tissue plate (BD Biosciences, CA, USA), 10 *G. mellonella* larvae were individually placed in wells and covered with 10% moist sand (white sand, Migros, Switzerland). Five infective juveniles were pipetted from either root-cap exudate or water solution and added to each well. 24-well plates were stored at 25°C for 3 days and *G. mellonella* larvae were dissected. The number of infected insect larvae was recorded.

#### EPN mobility 

EPN mobility was measured using a method modified after Tomalak, 2005. Into a 10-cm diameter Petri dish, we pipetted a 10-µl drop of 0.1% caffeine (99% pure, Alfa Aesar GmbH, Germany) on top of the 10ml of 2% solidified agar. About 100 EPNs from either root-cap exudate or water formulations were slowly pipetted onto a 5-mm diameter filter paper (cut out of bigger filter papers; Schleicher & Schuell GmbH, Germany). To facilitate removal of excessive water, the filter paper was laid on a paper towel. The filter was transferred immediately onto the drop of caffeine, with the EPNs on the upper side of the disc. The Petri dish was covered and stored in the dark at room temperature for 30min. As caffeine strongly repels EPNs, motile EPNs would quickly crawl away from this region onto the agar plate. After incubation, the disc was rinsed in a 1.5-ml centrifuge tube (Vaudaux-Eppendorf AG, Switzerland) containing water and the total number of sessile nematodes, recovered from the disc, was counted and the percentage of mobile EPNs calculated. This experiment was repeated five times per treatment.

#### Estimation of the neutral lipid content 

Estimation of the neutral lipid content was performed using a method modified after Patel *et al.*, 1997). About 50 infective juveniles were sampled from each formulation described above. They were poured into a glass vial (7ml amber glass vial; Suppelco, Sigma-Aldrich Chemie GmbH, Switzerland) and flooded with 70% ethanol saturated with Oil Red O (Sigma-Aldrich Chemie GmbH, Switzerland). The glass vials were incubated at 60°C for 20min. The excess of staining solution was removed before the addition of 5ml of glycerol:water (50:50 v/v) solution. EPNs were left to settle overnight at room temperature. After placing the EPNs on a slide, the lipid content of the stained nematodes was estimated by comparison with a neutral lipid index scale [from 8 (fully stained) to 1 (no staining): Patel *et al.*, 1997].

### Heat and cold stability of the green-pea exudate

The stability of green-pea exudate exposed to a temperature gradient was assessed. Aliquots of the exudate were either frozen (–80°C and –20°C) for 12h or heated up (20, 30, 40, 60, 80, and 100°C) for 10min. Controls consisted of water only, subjected to the same temperature treatments. For all treatments, induction of EPN quiescence was tested as described above and repeated seven times with two batches of EPNs.

### Dilution of root cap exudate and the effect on bioactivity

Following the procedure described in the previous sections, different concentrations of pea root-cap exudate were tested. Exudate from green pea cv. Lancet was collected and freeze dried. The resulting material was diluted in 1ml (original concentration), 1.25, 1.5, 1.75, 2, 3, and 4ml of water. Quiescence induction in *H. bacteriophora* was tested for each concentration; controls consisted of water only. The number of quiescent nematodes was recorded after 12h of induction. Each test was repeated 15 times.

Increasing activity of EPNs was observed with the reduction of the exudate concentration. Therefore, we assessed EPN activity more precisely by counting the number of oscillations per minute for individual *H. bacteriophora* after 12h exposure to a low concentration of pea root-cap exudate (in 1.5ml of water). Head oscillations of EPNs exposed to the original concentration of the exudate (in 1ml of water) and to water only were also recorded. This test was repeated with 10 different individuals from three different batches of nematodes.

Following the five-on-one methodology (see above and Grewal, 2005), the infectiousness of highly active EPNs in low concentration green pea exudate was compared to those in water only. In 24-well plates, 12 *G. mellonella* larvae were covered with 10% moist sand previously mixed with either 1.5× diluted green-pea exudate or water (9:1, v/v). Five infective juveniles of the EPN *H. bacteriophora* were individually transferred in each well. Plates were wrapped in Parafilm and incubated at 25°C in the dark. The number of EPN-infected larvae was recorded every 12h for 96h, starting 12h after EPN inoculation. This experiment was replicated four times with three different batches of EPNs.

The effect of the exudate concentrations was also tested on plant-parasitic nematodes. Following the procedure described above, the head oscillation counts per minute of the soybean cyst nematode *Heterodera glycine* Ichinohe (Tylenchida: Heteroderinae) and the root-knot nematode *Meloidogyne incognita* Kofoid and White (Tylenchida: Heteroderinae) were recorded in 1.5×-diluted exudate and in water.

### Statistical analyses

Statistical tests were conducted in SigmaPlot 12.1.0.15 (Systat Software GmbH Germany).

#### Quiescence induction by the root exudates of various green pea cultivars 

To determine differences in quiescence induced by the different cultivars we analysed the data using a one-way ANOVA procedure. Statistical differences within groups were evaluated using a Tukey post-hoc test.

#### Effect of root-cap exudate on the quality of EPNs over time 

EPN infectiousness, mobility, and neutral lipid content were tested using an RM two-way ANOVA procedure with treatment and time as factors. Statistical differences within groups were evaluated using a Tukey post-hoc test.

#### Heat and cold stability of the green pea exudate 

Differences in induced quiescence after temperature treatments were measured using a one-way ANOVA on ranks procedure. Statistical differences within groups were evaluated using a Tukey post-hoc test.

#### Dilution of root-cap exudate and the effect on bioactivity 

The effect of dilution on the induced quiescence to EPNs was tested using one-way ANOVA on ranks procedure. Statistical differences within groups were evaluated using a Tukey post-hoc test.

Increased activity was tested with a one-way ANOVA comparing the EPN head oscillations when exposed to both concentrations of green pea exudate or water. Infectiousness of EPNs exposed to either water or low-concentration exudate was tested using an RM two-way ANOVA procedure with treatment and time as factors. Statistical differences within groups were evaluated using a Tukey post-hoc test.

Differences between *H. glycine* and *M. incognita* head oscillations per minute recorded in water or in both concentrations of exudate were tested with a one-way ANOVA and a one-way ANOVA on ranks, respectively.

## Results

### Exudate-induced quiescence and recovery of EPNs

Each EPN species that experienced contact with the green pea root exudate exhibited a state of quiescence (Table 1). Recovery was complete for *S. carpocapsae*, *H. megidis*, and *H. bacteriophora*. A large majority of *S. feltiae* recovered from quiescence. Each EPN species was still infectious after recovering from induced quiescence (Table 1).

**Table 1.** Exudate-induced quiescence, recovery, and subsequent infectiousness of three EPN species

	Quiescence (%)	Recovery (%)	Infectiousness
*Steinernema feltia*	91±6	57±12	+^a^
*S. carpocapsae*	93±4	100	+
*Heterorhabditis megidis*	89±9	97±2	+
*H. bacteriophora*	95±2	100	+

^a^Some individuals remained quiescent after removal of the exudate. Nonetheless, larvae treated with recovered *S. feltia* were infected.

### Quiescence induction by the exudates of various green-pea cultivars

**Fig. 1.**
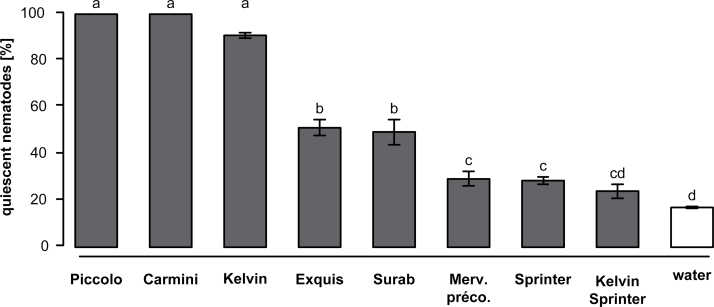
Induced quiescence in *Heterorhabditis bacteriophora* varies among green pea cultivars. Induced EPN quiescence was significantly influenced by the cultivar the root-cap exudates were collected from (grey bars). Three cultivars induced quiescence close or equal to 100% whereas some barely reached 30%, but were still different from the percentage of inactive nematodes recorded in water (white bar). One cultivar (Kelvin Sprinter) was not different from water. Letters indicate statistical differences. Bars represent SEM.

The induction of quiescence in *H. bacteriophora* was highly dependent on the green-pea genotypes (Fig. 1, ANOVA, F_8,93_ = 26.686, *P* ≤ 0.001). Exudate from cv. Kelvin Sprinter had the lowest induction rate of quiescence (~25%), whereas the exudate of some cultivars resulted in almost 100% quiescence (i.e. cv. Picolo) (Fig. 1).

### Effect of root-cap exudate on the quality of EPNs over time

**Fig. 2.**
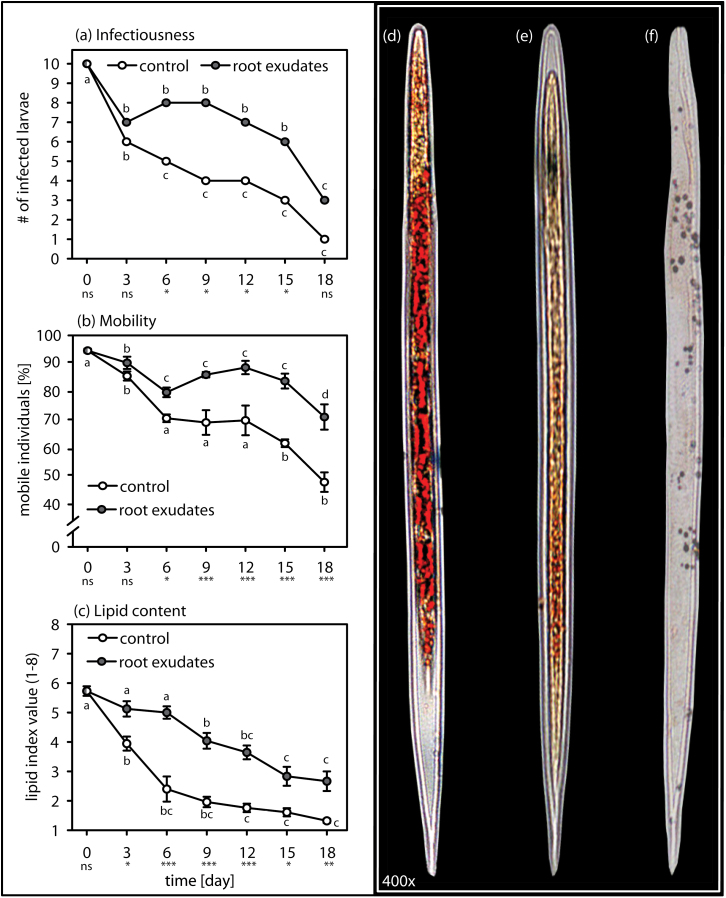
Induced quiescence enhances over time storage of the EPN *Heterorhabditis bacteriophora*. Quality traits were better conserved over time when EPNs were stored in a solution with root-cap exudates from green pea cv. Lancet (grey circle) as compared to EPNs stored in water (white circle). (A) EPN infectiousness remained significantly higher in root-cap exudates as compared to water during most of the experiment. (B) When stored in root-cap exudates, EPNs stayed mobile for longer compared to the EPNs in water over most of the duration of the experiment. (C) Because EPNs stored in root-cap exudate were quiescent, their lipid reserves were maintained at a significantly higher level over time compared to lipid reserves measured in EPNs stored in water. (D–F) After being stained with Oil Red O, lipids appear in red inside the nematode body. A visual assessment allows an evaluation of the lipid content according to the 1–8 lipid index (Patel *et al.*, 1997). According to this index, (D) fully stained EPNs were given an 8, (E) intermediately stained EPNs received a 4, and (F) individuals without staining were recorded as 1. Level of significance between treatments on one day are indicated as not significant (ns), *P* < 0.05 (*), *P* ≤ 0.01 (**), *P* ≤ 0.001 (***). Statistical differences within treatments over time are noted with letters. Bars indicate SEM.

Induced quiescence in *H. bacteriophora* helped to maintain its performance over time (Fig. 2A, B, C). Overall, infectiousness of *H. bacteriophora* that had been stored in exudate was higher than for EPNs that had been stored in water (Fig. 2A; two-way RM ANOVA, *F*_*1,139*_ = 12.05, *P* ≤ 0.001). After recovering from quiescence, EPNs that had been stored in exudate were significantly more mobile than EPNs that had been stored in water (Fig. 2A; two-way RM ANOVA, *F*_*1,139*_ = 66.839, *P* ≤ 0.001) and this remained significantly higher over the 18 days of the experiment (Fig. 2B; two-way RM ANOVA, *F*_*6,139*_ = 3.845, *P =* 0.003). Lipid reserves in quiescent nematodes were significantly higher than in EPNs stored in water (Fig. 2C; two-way RM ANOVA, *F*_*1,139*_ = 53.129, *P* ≤ 0.001) and remained so over the course of the experiment (Fig. 2C; two-way RM ANOVA, *F*_*6,139*_ = 2.414, *P* = 0.03).

### Heat and cold stability of the green-pea exudate

Exposing the exudate to different temperatures did not impair its bioactivity, and quiescence induction levels were similar to those measured in the control exudate. After heating or freezing, the exudate still induced quiescence in exposed *H. bacteriophora*, which was not the case for the water control [Supplementary Figure S1 (heat: ANOVA on ranks, *H* = 45.419, *P* < 0.001); Supplementary Figure S2 (cold: ANOVA on ranks, *H* = 33.289, *P* ≤ 0.001)].

### Dilution of root-cap exudate and the effect on bioactivity

**Fig. 3.**
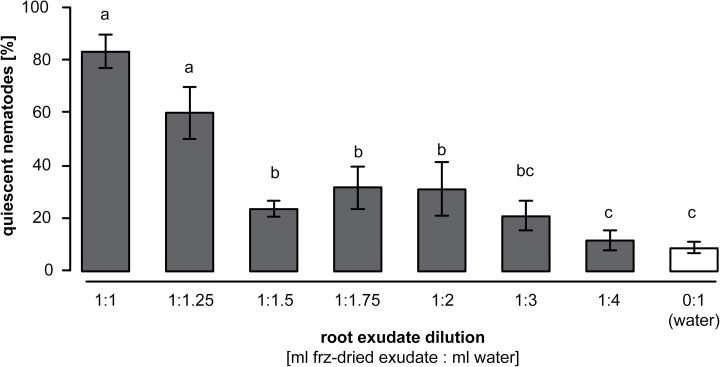
Level of induced quiescence in EPNs depends on root-cap exudate concentration. Lowering the concentration of the root-cap exudate reduced the induction of quiescence in the EPN *Heterorhabditis bacteriophora*. When freeze-dried exudates were diluted in 1.5× more water than its original concentration, the quiescence was reduced 4-fold compared to the original concentration. Low concentrations of exudates did not induce more quiescence than water. Letters indicate statistical differences. Bars indicate SEM.

**Fig. 4.**
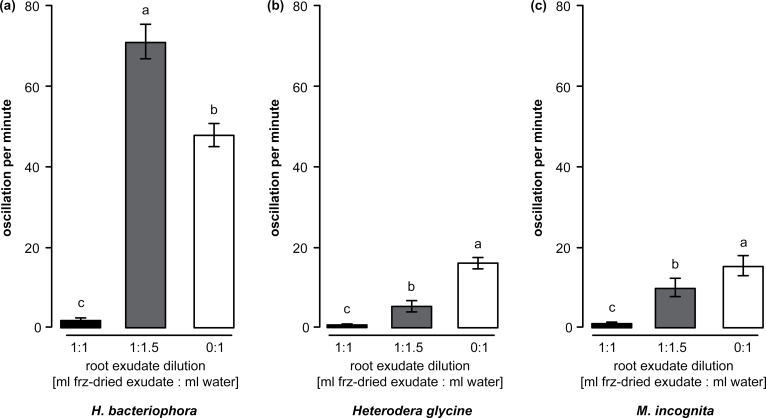
Low root-exudate concentration has a dual effect on nematode activity. (A) The number of oscillations per minute was significantly increased when the EPN *Heterorhabditis bacteriophora* was exposed to 1.5×-diluted exudates (grey bar) as compared to those in water (white bar). The original (1×) concentration of exudate induced almost complete quiescence in the exposed EPN. (B) The plant-parasitic nematode *Heterodera glycine* oscillation counts were higher in diluted exudate as compared to the original concentration. Whereas quiescence induction was incomplete at low concentration, the plant-parasitic nematode oscillation activity was still almost 3-fold lower than observed in water alone. (C) A similar pattern was observed for the plant-parasitic nematode *Meloidogyne incognita*. Letters indicate statistical differences. Bars indicate SEM.

**Fig. 5.**
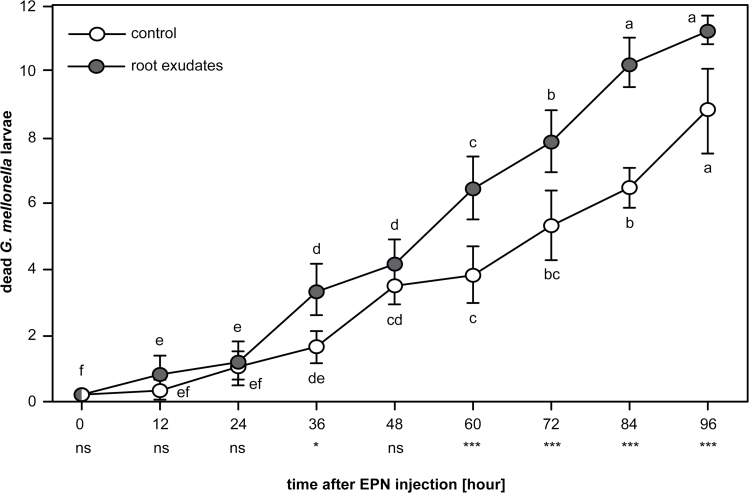
Low concentrations of exudate boosted the infectiousness of the EPN *Heterorhabditis bacteriophora*. EPNs in low concentrations of pea root-cap exudate (grey circles) were quicker and superior in infecting *Galleria mellonella* larvae than those in water alone (white circles). Levels of significance between treatments on one day are indicated as not significant (ns), *P* < 0.05 (*), *P* ≤ 0.01 (**), *P* ≤ 0.001 (***). Statistical differences within treatment over time are noted with letters. Bars indicate SEM.

Reducing the exudate concentration quickly reached a threshold beyond which induction of quiescence in *H. bacteriophora* failed (Fig. 3; ANOVA on ranks, *H*_*7,15*_ = 52.42, *P* ≤ 0.001), but instead resulted in increased movement (Fig. 4A; ANOVA, *F*_*2–89*_ = 491.427, *P* ≤ 0.001). At a low concentration, the exudate had a positive impact on the infectiousness of *H. bacteriophora* (Fig. 5; RM two-way ANOVA, *F*_*1,107*_ = 20.991, *P* = 0.006), and EPN exposed to low concentration exudates were more effective at killing *G. mellonella* over time as compared to EPN exposed to water only (Fig. 5; RM two-way ANOVA, *F*_*8,107*_ = 5.88, *P* ≤ 0.001). Contrary to the effect on EPNs, the same low concentration of exudate significantly reduced the mobility of the tested plant-parasitic nematode species *H. glycine* (Fig. 4B; ANOVA, *F*_*2–89*_ = 225.023, *P* ≤ 0.001) and *M. incognita* (Fig. 4C; ANOVA on ranks, *H* = 67.876, *P* ≤ 0.001).

## Discussion

Earlier observations had revealed that green pea root-cap exudate causes a loss of mobility and induces a state of quiescence in several plant-parasitic nematode species (Zhao *et al.*, 2000; Hubbard *et al.*, 2005), as well as in the bacteria-feeding nematode *Cenorhabditis elegans* Maupas (Zhao *et al.*, 2000; Hubbard *et al.*, 2005;), and the EPN *S. feltiae* (Hubbard *et al.*, 2005). The present study demonstrates that the activity of other EPN species also drops dramatically when exposed to the green pea root-cap exudate (Table 1). Quiescence is usually a reversible response in nematodes to toxic or unfavourable environmental conditions (Evans and Perry, 1976). As plant-parasitic nematodes, as well as other pathogens, preferably choose the elongation zone right behind the root tip to penetrate root tissues (Prot, 1980; Curl and Truelove, 1986; Gunawardena and Hawes, 2002), the induction of quiescence in root pathogens around this susceptible region has been interpreted as a defensive mechanism (Hawes *et al.*, 2000; Zhao *et al.*, 2000; Wuyts *et al.*, 2006). Recent findings showed that plant histone-linked extracellular DNA (exDNA) might be involved in this putative root tip defence (Wen *et al.*, 2009; Hawes *et al.*, 2011; Hawes *et al.*, 2012;). Functioning as an extracellular trap attracting and immobilizing pathogens (Hawes *et al.*, 2012), exDNA has been shown to be a key component in plant resistance to infection by pathogenic fungi (Wen *et al.*, 2009). By reducing or inhibiting the motility of these pathogens, roots can grow away from the threat, avoiding damage to their apical tips, while offering less vulnerable regions to herbivores. Interestingly, this is in contradiction to the common dogma that plants cannot escape a threat by moving away. Indeed, in addition to immobilizing pathogens (Hawes *et al.*, 2000), roots can potentially grow through a cumulative total of 6 m of soil within a day (Lynch and van Beem, 1993). This provides the roots with an escape strategy that is comparable to animals fleeing unfavourable biotic and abiotic conditions. To determine whether this strategy may indeed be effective against the relatively large and mobile nematodes requires additional experiments. The present study shows that the exudates involved in this potential defence mechanism not only affects the roots’ ‘foes’ but can also momentarily impair beneficial microorganisms such as EPNs (Table 1).

As with other plant defence traits, the quiescence potency of root exudates varies a lot among plant families (Hubbard *et al.*, 2005), but we also found dissimilarities among genotypes of a particular species (Fig. 1). The results presented here provide a new example of how plant breeding can affect the concentrations of secondary metabolites that are important for plant performance (e.g. Hubbard *et al.*, 2005; Rasmann *et al.*, 2005; Köllner *et al.*, 2008; Hiltpold *et al.*, 2010c; Erb *et al.*, 2011; Robert *et al.*, 2012; Meihls *et al.*, 2013).

Induced quiescence in EPNs may have interesting applications in biological control. Indeed, EPN mass production has been optimized (Ehlers, 2001), but downstream processes such as storage and transport can still be problematic. Inducing quiescence in EPNs with the root exudate significantly maintained EPN performance traits such as infectiousness, mobility, and lipid content (Fig. 2A–C). As in insect parasitoids (Casas *et al.*, 2005; Denis *et al.*, 2013), EPNs rely on their lipid reserves while foraging and infecting insect hosts (Patel *et al.*, 1997). Loss of lipids was significantly lower in quiescent *H. bacteriophora* than in EPNs stored in water (Fig. 2C), possibly explaining their higher mobility and infectiousness after recovery (Fig. 2A and B). Similar to root penetration by plant-parasitic nematodes (Hubbard *et al.*, 2005), infectiousness and mobility of EPNs recovering from quiescence tended to increase (Fig. 2A and B), suggesting a general increase of nematode activity after quiescence recovery. Whereas the timescale used in this study is not pertinent for long-term storage, these data clearly imply that the addition of the active compounds to the storage formulation will increase the EPN shelf life.

Induced quiescence in EPNs can be problematic when aiming at controlling insect pests in the rhizosphere. However, this apparent biological conflict might not occur in natural environments. Indeed, at lower concentrations of the exudate, as can be expected in the field, EPN activity was significantly boosted, while plant-parasitic nematodes remained quiescent (Fig. 4). Further tests under natural conditions must be performed, but the enhanced infectiousness of ‘frenetic’ EPNs (Fig. 5) suggests a dual effect of the tested exudate: root defences can simultaneously subdue ‘foes’, and invigorate ‘friends’.

The present study reinforces the idea that plant exudates can help shape the dynamics of interactions not only in the rhizosphere, but also at more distant regions in the soil (Rasmann *et al.*, 2012*b*; Erb *et al.*, 2013; Hiltpold *et al.*, 2013;). The strategy of plants to protect their root tips with exudates is in apparent conflict with an alternative strategy, the attraction of natural enemies upon herbivorous attack. Yet, because exudate bioactivity seems tuned to have opposite effects on plant mutualists and antagonists, the two strategies might effectively complement each other. The complexity of biotic and abiotic interactions in the rhizosphere is huge and difficult to fully understand, but each new finding leads to a better and broader understanding of this very particular ecosystem. Therefore the prospect of harnessing root exudation and exploiting it for enhanced pest control (Hiltpold and Turlings, 2012) may indeed be realistic (Degenhardt *et al.*, 2009; Hiltpold *et al.*, 2010a, b; Ali *et al.*, 2012; Hiltpold *et al.*, 2012).

## Supplementary material

Supplementary data can be found at *JXB* online.

Supplementary Figure S1. Effect of heat on the bioactivity of the pea root-cap exudate and the induction of quiescence in *H. bacteriophora*.

Supplementary Figure S2. Induction of quiescence in *H. bacteriophora* after the exudate was frozen.

## Funding

Our work in this field is supported by a Swiss economic stimulus grant awarded to the National Centre of Competence in Research (NCCR), Plant Survival, as well as by a postdoctoral fellowship, PBNEP3-13485, from the Swiss National Science Foundation awarded to IH.

## Supplementary Material

Supplementary Data
