# Supplementary material for: The dual effects of root-cap exudates on nematodes: from quiescence in plant-parasitic nematodes to frenzy in entomopathogenic nematodes
Source: J Exp Bot. 2014 Aug 27;66(2):603–11. doi: 10.1093/jxb/eru345 (PMC4286403; doi:10.1093/jxb/eru345)
Supplement: Supplementary Data [file supp_66_2_603__index.html]

The dual effects of root-cap exudates on nematodes: from quiescence in plant-parasitic nematodes to frenzy in entomopathogenic nematodes — The dual effects of root-cap exudates on nematodes: from quiescence in plant-parasitic nematodes to frenzy in entomopathogenic nematodes — Supplementary Data 

# The dual effects of root-cap exudates on nematodes: from quiescence in plant-parasitic nematodes to frenzy in entomopathogenic nematodes

## Supplementary Data

Data files

**Files in this Data Supplement:**

- Supplementary Data - Supplementary Data
