# Supplementary material for: The dual effects of root-cap exudates on nematodes: from quiescence in plant-parasitic nematodes to frenzy in entomopathogenic nematodes
Source: J Exp Bot. 2014 Aug 27;66(2):603–11. doi: 10.1093/jxb/eru345 (PMC4286403; doi:10.1093/jxb/eru345)
Supplement: Supplementary Data [file supp_eru345_jexbot129270_file001.pdf]

The dual effects of root cap exudates on nematodes: from quiescence in plant parasitic nematodes to frenzy in entomopathogenic nematode

Ivan Hiltpold[1], Geoffrey Jaffuel[2], Ted C.J. Turlings[2]

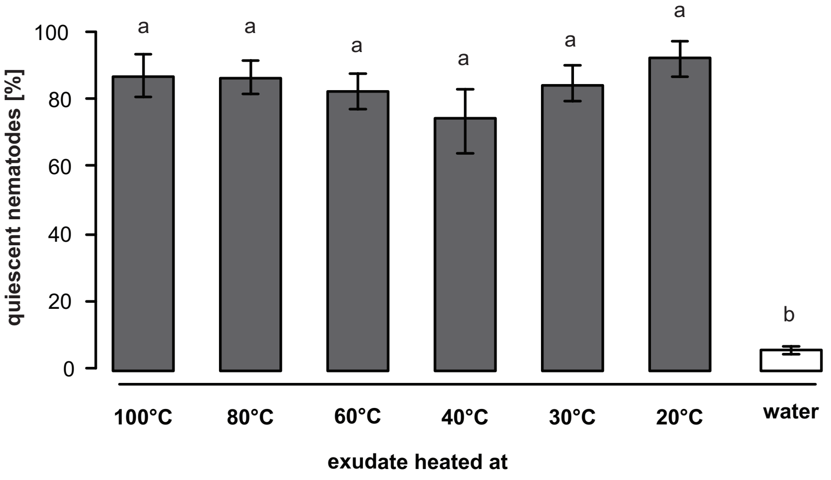

Supplementary figure S1

Heating the green pea exudate did not impair the induction quiescence in *Heterorhabditis bacteriophora* (grey bars), whereas inactive nematode percentage in water (white bar) remained very low. Letters indicate statistical differences. Bars represent SEM.

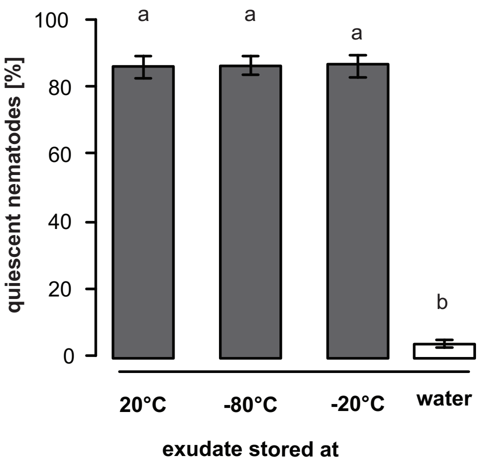

Supplementary figure S1

Freezing the green pea exudate did not impair the induction quiescence in *Heterorhabditis bacteriophora* (grey bars), whereas inactive nematode percentage in water (white bar) remained very low. Letters indicate statistical differences. Bars represent SEM.
